# NKT Cells Stimulated by Long Fatty Acyl Chain Sulfatides Significantly Reduces the Incidence of Type 1 Diabetes in Nonobese Diabetic Mice

**DOI:** 10.1371/journal.pone.0037771

**Published:** 2012-05-23

**Authors:** Lakshmimathy Subramanian, Hartley Blumenfeld, Robert Tohn, Dalam Ly, Carlos Aguilera, Igor Maricic, Jan-Eric Mansson, Karsten Buschard, Vipin Kumar, Terry L. Delovitch

**Affiliations:** 1 Laboratory of Autoimmune Diabetes, Robarts Research Institute, Western University, London, Ontario, Canada; 2 Department of Microbiology and Immunology, Western University, London, Ontario, Canada; 3 Harvard Medical School, Brigham and Women’s Hospital, Boston, Massachusetts, United States of America; 4 Laboratory of Autoimmunity, Torrey Pines Institute for Molecular Studies, San Diego, California, United States of America; 5 Department of Neuroscience, Sahlgrenska University Hospital, Mölndal, Sweden; 6 Bartholin Institute, Rigshospitalet, Copenhagen, Denmark; Karolinska Institutet, Sweden

## Abstract

Sulfatide-reactive type II NKT cells have been shown to regulate autoimmunity and anti-tumor immunity. Although, two major isoforms of sulfatide, C16:0 and C24:0, are enriched in the pancreas, their relative role in autoimmune diabetes is not known. Here, we report that sulfatide/CD1d-tetramer^+^ cells accumulate in the draining pancreatic lymph nodes, and that treatment of NOD mice with sulfatide or C24:0 was more efficient than C16:0 in stimulating the NKT cell-mediated transfer of a delay in onset from T1D into NOD.*Scid* recipients. Using NOD.CD1d^−/−^ mice, we show that this delay of T1D is CD1d-dependent. Interestingly, the latter delay or protection from T1D is associated with the enhanced secretion of IL-10 rather than IFN-g by C24:0-treated CD4^+^ T cells and the deviation of the islet-reactive diabetogenic T cell response. Both C16:0 and C24:0 sulfatide isoforms are unable to activate and expand type I iNKT cells. Collectively, these data suggest that C24:0 stimulated type II NKT cells may regulate protection from T1D by activating DCs to secrete IL-10 and suppress the activation and expansion of type I iNKT cells and diabetogenic T cells. Our results raise the possibility that C24:0 may be used therapeutically to delay the onset and protect from T1D in humans.

## Introduction

Sulfatide (3′-sulfated b-galactosylceramide), a glycosphingolipid expressed primarily in ceramide and negatively charged sulfate moieties. The ceramide galactosyl sulfotransferase enzyme adds a sulfate group to galactosylceramide (GalCer) to produce sulfatide in the trans-golgi apparatus. Arylsulfatase A degrades sulfatide to GalCer, and in pancreatic islet beta cells GalCer is recycled back to the cis-Golgi apparatus [1]. While the ratio of the amount of C24:0 sulfatide to C16:0 sulfatide is ≥5∶1 in the brain, equivalent amounts of C16:0 and C24:0 sulfatide are found in the pancreas [1]. In addition, the ratio of the amount of C24:0 sulfatide to C24:1 sulfatide is 3∶1 in pancreatic islets [1].

Sulfatide binds to MHC class I-like CD1 group 1 (CD1a, CD1b, CD1c) and group 2 (CD1d) glycoproteins and presented to T cells [2]. The CD1d-reactive type I invariant NKT (iNKT) cells express a semi-invariant TCR comprised of Va14-Ja18 preferentially paired with Vb8.2, 7, 2 in mice or Va24-Ja18 paired to Vb11 in humans [3], [4]. Type II NKT cells are also CD1d-restricted but contain a diverse TCR repertoire [5]. While the prototypic synthetic glycolipid KRN7000, a structurally similar form of aGalCer C26:0 originally derived from a marine sponge [3], activates only type I iNKT cells [3], [4], sulfatide is recognized by a major subset of type II NKT cells [2], [5], [6].

The conformation of the A’ and F’ channels of CD1d differs between their antigen-bound and antigen-unbound states [7]. The A’ channel accommodates the sphingosine side chain (18 carbon atoms) of a sphingoglycolipid, whereas the fatty acyl chain occupies the F’ channel (26 carbon atoms). Since structural differences enable aGalCer and sulfatide to differ in their mode of binding to CD1d, these two sphingoglycolipids result in different T cell activation and signaling pathways [8]. Upon binding to CD1d, sulfatide exposes more of its lipid group than aGalCer, which may lead to differential iNKT cell activation and Th1- or Th2-type cytokine secretion depending on the length of the fatty acyl chain and a spacer lipid(s) bound in the CD1d groove [9], [10].

Type 1 diabetes (T1D) is an autoimmune disease that results from the selective T cell-mediated destruction of insulin-producing pancreatic islet beta cells. T1D is commonly studied in nonobese diabetic (NOD) mice, which spontaneously develop T1D due in part to functional and numerical deficiencies in Treg cells and iNKT cells [3]. Administration of glycolipid antigens (e.g. aGalCer) can activate iNKT cells and restore this iNKT cell deficiency, and thereby protect against T1D in NOD mice [11]–[14]. The mechanism of protection may involve crosstalk between many immune cell types, including iNKT cells and regulatory T (Treg) cells [15], [16]. A deficiency in Treg activity may exacerbate T1D, as iNKT cell activation results in increased secretion of inflammatory cytokines as well as the transactivation of B, T and NK cells [3], [4]. In addition, a polarized Th2 shift towards IL-4 production was reported to mediate protection from T1D [17].

A rapidly increasing role for the regulation of various states of inflammation and disease by type II NKT cells is emerging, as is evident from recent experimental studies of the amelioration of autoimmune disorders such as EAE [2] and T1D [18], tumour immunity [6], experimental hepatitis [19], HIV infection [20] and hepatic ischemic reperfusion injury [21] by treatment with sulfatide. Indeed, in T1D, non-classical CD69^–^CD49^high^ NKT cells that proliferate in response to sulfatide can protect against T1D in NOD mice [18].

Recently, we identified a novel pathway of immune regulation in which sulfatide-activation of type II NKT cells results in anergy induction in type I NKT cells [19]–[22]. Importantly, during this NKT-DC interaction, DCs are also tolerized, thus further inhibiting the protein-reactive CD4^+^ effector T cells mediating autoimmunity. Sulfatide-mediated regulation of type I NKT cells not only plays an important role in the control of autoimmune diseases, but also has a major effect in immune surveillance of tumors [19], [20]. In this report, we examined whether long fatty acyl chain sulfatide-mediated activation of type II NKT cells can protect NOD mice from diabetes. In addition, we further examined whether the protection results in the modulation of potentially pathogenic T cells reactive to islet beta cell antigens.

Type 2 diabetes patients have low serum levels of sulfatide [23], and Type 2 diabetes susceptible ob/ob and db/db mice are deficient in C16:0 sulfatide but not C24:0 sulfatide [24]. In contrast, both sulfatide isoforms are present in NOD mice and human pancreas [25]. C16:0 sulfatide is one of the first non-protein chaperones described, as it mediates the folding and preservation of insulin crystals [26]. Sulfatide also facilitates pancreatic beta cell rest by modulation of ATP-sensitive potassium channels [27] and by reducing the secretion of pro-inflammatory cytokines such as IFN-g and TNF-α [28].

Although treatment of NOD mice with brain sulfatide protects them from T1D [29], it is not known whether one or both sulfatide isoforms elicits this protection. Therefore, identification of the sulfatide isoform that can protect from T1D is important, as it may have future implications for the prevention and therapy of T1D in humans. A priori, it has been suggested that the longer C24:0 sulfatide isoform may be more effective in protection from T1D due to its more efficient binding to CD1d [9]. In this study, we analyzed the therapeutic capacity of the C16:0 and C24:0 sulfatide isoforms to protect the transfer of T1D by lymphocytes from donor NOD mice to recipient immunodeficient NOD.*Scid* mice. The ability of these isoforms to activate NKT cells and modulate their cytokine secretion profiles were also investigated. Our results demonstrate that treatment of NOD mice with C24:0 sulfatide preferentially protects against the transfer of T1D, and suggest that this isoform may have therapeutic value in clinical trials in human subjects at risk for T1D and/or in patients newly diagnosed with T1D.

## Results

### C24:1 and C24:0 Sulfatide Protect NOD Mice from T1D

**Figure 1:**
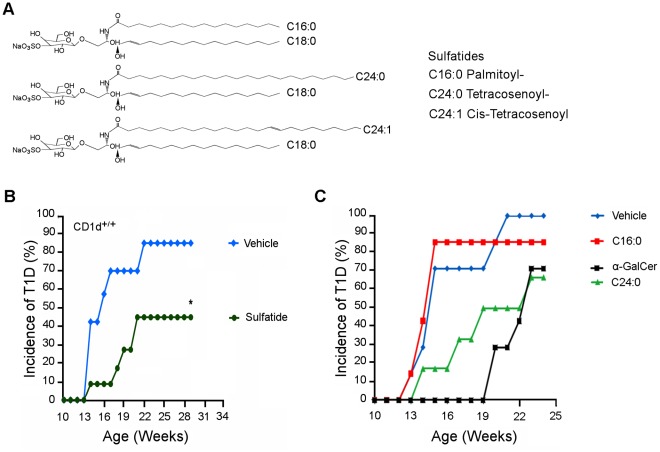
Structure-function analyses of sulfatides. (**A**) Structures of the C16:0, C24:0 and C24:1 sulfatide isoforms. (**B**) Treatment with sulfatide reduces the incidence of spontaneous T1D in NOD mice. Female NOD wild type (CD1d^+/+^) mice (12 week-old, n = 10–12/group) were injected once weekly for 3 weeks with 20 mg of either sulfatide, GM1 or PBS-vehicle until 15 weeks of age. The P value between the values in the control (PBS/vehicle or mGM1) versus sulfatide group was <0.0001. The occurrence of spontaneous T1D was followed for up to 30–34 weeks of age by measuring BGL. Two consecutive BGL readings of >250 mg/dl was considered diabetic. These data are representative of 4 independent experiments. (**C**) Treatment of NOD mice with C24:0 but not C16:0 sulfatide reduces the lymphocyte-mediated adoptive transfer of T1D. Female NOD mice (3–5 week-old, n = 10/group) were injected i.p. with either control vehicle (PBS), aGalCer (4 mg/dose) or sulfatide (C16:0 or C24:0, 100 mg/dose) on day 0 and day 4. Pooled splenocytes and PLN lymphocytes (2×10^7^) were transferred to female NOD.*Scid* recipients, and the recipient mice were monitored until 24 weeks of age for the development of T1D by determining their BGL. Results shown are representative of 3 independent and reproducible experiments.

Initially, we determined whether the synthetic isoforms of sulfatide can protect NOD mice from the spontaneous development of T1D. The structures of the C16:0, C24:0 and C24:1 sulfatide isoforms are shown in Fig. 1A. C24:1 sulfatide was included in this study since it forms a stable complex with CD1d, can assemble into tetrameric complexes [9] and treatment of B10.PL, C5BL/6 and SJL/J mice with C24:1 but not sulfatide with shorter fatty acyl side chains reverses ongoing experimental allergic encephalomyelitis (EAE) [*Maricic et al*., *in preparation*], a mouse model of multiple sclerosis [5]. Treatment of female NOD mice with either C16:0 or C24:0 sulfatide according to our previously described [30], [31] multi-dose protocol used for aGalCer or its analogs (i.p. injection, every other day for 3 weeks with a 4 mg/dose of glycolipid or vehicle) did not protect the mice from T1D (our unpublished data). As our preliminary experiments indicated that aGalCer provides a much stronger stimulus of CD4^+^ T cell proliferation than C16:0 and C24:0, we injected higher doses (50 mg/dose or 100 mg/dose) of C16:0 and C24:0 into NOD mice according to the same multi-dose protocol and still found that these higher doses provided only very low (10–20%) protection of the mice from the spontaneous development of T1D. In contrast, when NOD female mice (12 week-old, 10–12 mice/group) were administered three weekly injections (i.p.) of 20 mg of sulfatide, significant protection of these NOD mice from T1D was achieved (Fig. 1B). Similar results were obtained for NOD male mice. Thus, the amount of protection of NOD mice from T1D seems to vary with the particular dose and frequency of sulfatide used for treatment.

Previously, it was reported that the administration of brain-derived sulfatide to NOD mice inhibits the ability of lymphocytes from these donor mice to transfer T1D to irradiated young NOD recipient mice [29]. Therefore, we next examined whether treatment of NOD mice with the C16:0 or C24:0 isoform of sulfatide induces a similar protection from the lymphocyte-mediated adoptive transfer of T1D. Female NOD mice were injected i.p. with two doses (100 mg/dose) of either C16:0 sulfatide, C24:0 sulfatide, αGalCer or vehicle on day 0 and day 4, as reported [29], [30]. Pooled spleen- and PLN-derived lymphocytes were transferred to female immunodeficient (no lymphocytes) NOD.*Scid* mice, and the recipient mice were monitored for the development of T1D by determining their blood glucose levels (BGL). Compared to C16:0, C24:0 and aGalCer both delayed the onset of T1D appreciably in recipient NOD.*Scid* mice, with aGalCer yielding the longest delay (Fig. 1C). Moreover, by 24 weeks of age, treatment with either C16:0 sulfatide or control vehicle yielded a high incidence of disease (approximately 90–100% incidence of T1D), whereas C24:0 sulfatide or aGalCer treatments each yielded only about 65–70% incidence of T1D. Thus, relative to the control vehicle, C24:0 but not C16:0 sulfatide delays onset of T1D and protects (P<0.05) against the adoptive transfer of T1D. Our preliminary data further suggest that administration of 20 mg of C24:1 sulfatide every three weeks also significantly protects female NOD mice from T1D.

### Capacity of Sulfatide Isoforms to Protect from T1D is CD1d-dependent

**Figure 2:**
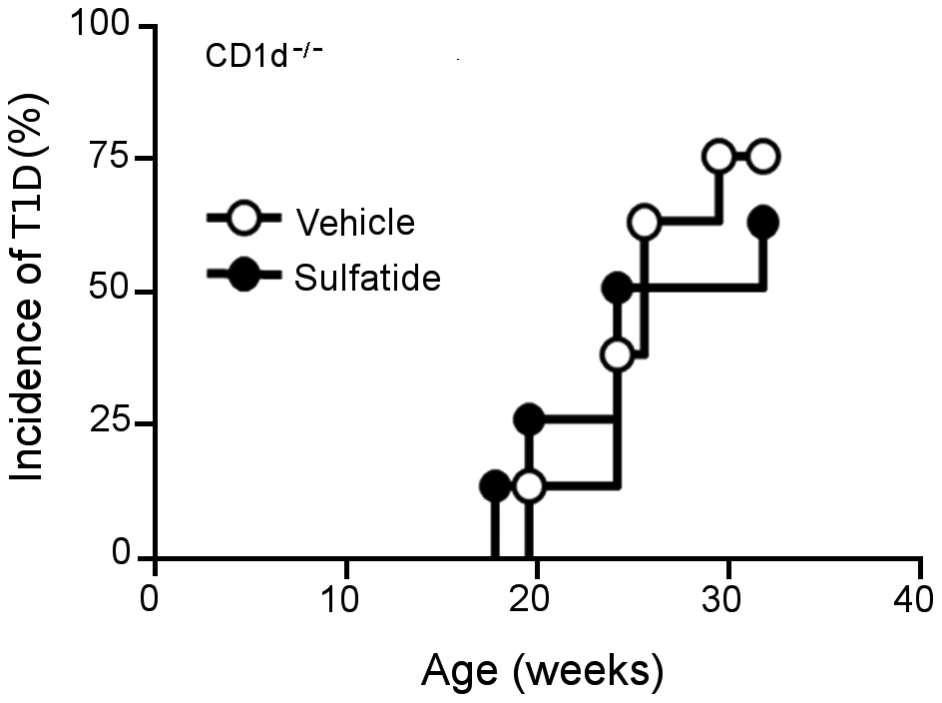
C24:0 sulfatide-induced protection from the adoptive transfer of T1D is CD1d-dependent. Female NOD.CD1d^−/−^ mice (3–5 week-old, n = 10/group) were injected i.p. with either control vehicle (PBS) or C24:0 sulfatide (100 mg/dose) on day 0 and day 4. Pooled splenocytes and PLN lymphocytes (2×10^7^) were transferred to female NOD.*Scid* recipients, and the recipient mice were monitored until 32 weeks of age for the development of T1D by determining their BGL. Two consecutive BGL readings of >250 mg/dl was considered diabetic. Data shown represent one of two representative and reproducible experiments.

The following experiment was conducted to determine whether the protection from T1D induced by sulfatide or the transfer of T1D elicited by C24:0 depends on the expression of CD1d and presence of iNKT cells. Using CD1d-deficient mice that lack the expression of CD1d and presence of iNKT cells, we observed a 90–95% incidence of T1D in NOD.*Scid* recipients of lymphocytes from donor NOD.CD1d^−/−^ mice treated with C24:0 sulfatide or vehicle (Fig. 2), in support of the reports that splenocytes from B6.CD1d^−/−^ mice are unable to function as APCs [30] or present sulfatides to human type II NKT cells [32]. This incidence was significantly greater than the 70% incidence of T1D found in NOD.*Scid* recipients of lymphocytes transferred from C24:0 treated wild-type NOD mice. Thus, the capacity of C24:0 sulfatide to protect against the transfer of T1D is dependent on the expression of CD1d and presence of iNKT cells in the lymphocyte population that transfers this protection. Our results demonstrate that C24:0 sulfatide treatment delays the onset of T1D in NOD mice in a CD1d-dependent manner.

### Ability of Sulfatide Isoforms to Protect from T1D Correlates with its Capacity to Stimulate a Spleen CD4^+^ T cell Proliferative Response

**Figure 3:**
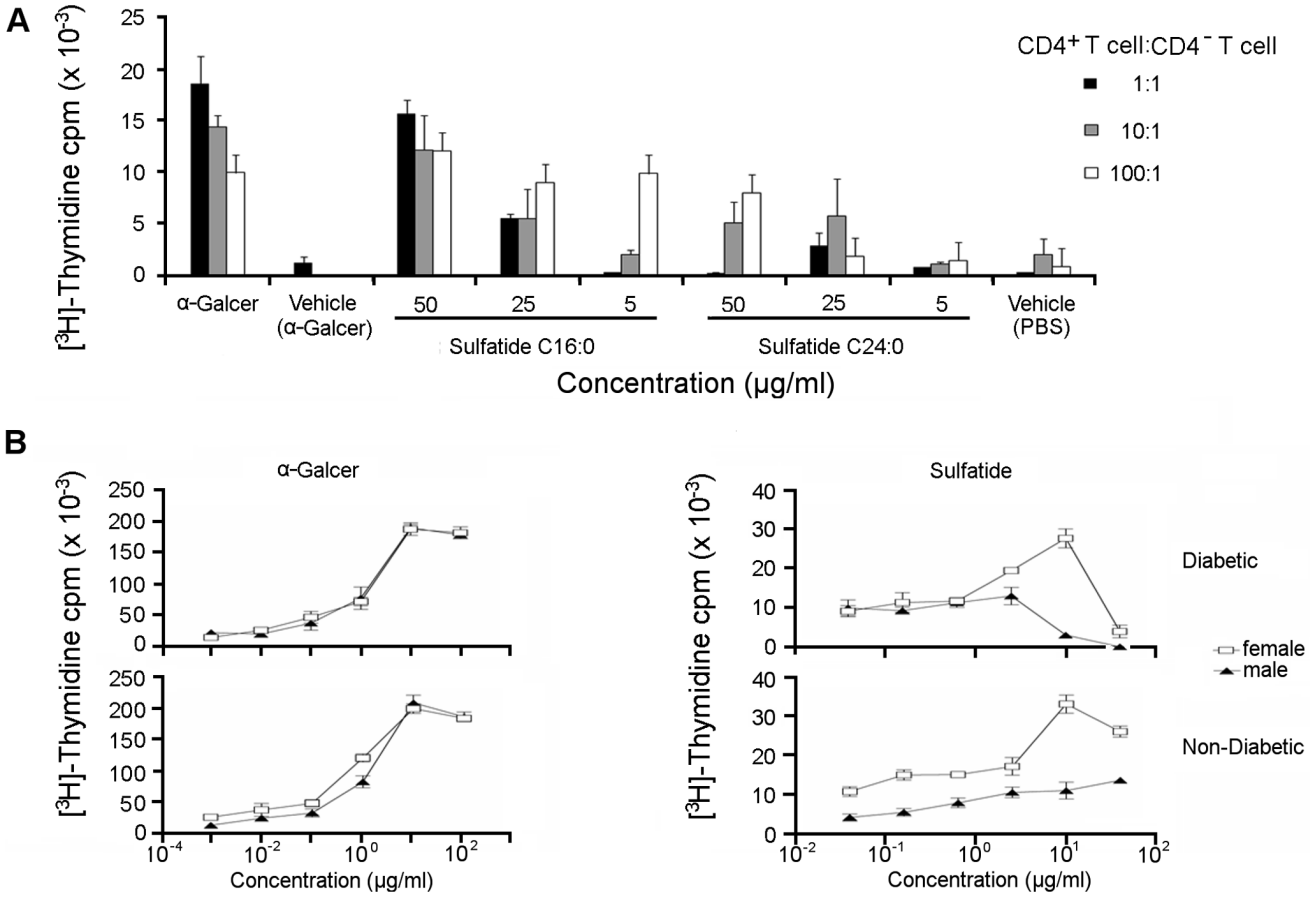
Analyses of NOD T cell proliferative responses stimulated by sulfatides. (**A**) Ability of C24:0 sulfatide to protect from T1D does not correlate with its capacity to stimulate a CD4^+^ T cell proliferative response. Spleen CD4^+^ T cells from female NOD mice (3–5 week-old) were co-cultured for 72 h *in vitro* in the presence of mitomycin C treated CD4^–^ cells at CD4^+^:CD4^–^ ratios of 1∶1, 10∶1 or 100∶1 with either control vehicle, aGalCer (100 ng/ml) or varying concentrations (5–50 mg/ml) of sulfatide (C16:0 or C24:0). [^3^H]-thymidine (1 mCi/well) was added for the final 18 h of culture before harvesting the cells and quantitating their thymidine incorporation. Data shown were obtained from one of two representative and reproducible experiments. (**B**) Proliferative responses of splenocytes from non-diabetic and diabetic NOD mice (2–4 mice/group) in response to *in vitro* stimulation by sulfatide or aGalCer. Splenocytes (8×10^5^) were incubated in a 96-well plate with a titrated concentration of sulfatide or aGalCer, and [^3^H]-thymidine incorporation in triplicate wells was determined following culture for 90 h, as previously described [2]. No detectable response was found to another control glycolipid mGM1 in these assays. Data from one of three representative experiments are shown.

To better understand why C24:0 but not C16:0 protects from the transfer of T1D, we initially analyzed the relative capacity of these sulfatide isoforms to stimulate the *in vitro* proliferation of CD4^+^ T cells. The proliferative responses of NOD splenic CD4^+^ T cells to either control vehicle, aGalCer (100 ng/ml) or varying doses (5–50 mg/ml) of C16:0 or C24:0 in the presence of different ratios of mitomycin C treated CD4^–^ T cells (CD4^+^ T cells:CD4^–^ T cells = 1∶1, 10:1 or 100:1) were measured after 72 h. The CD4^–^ T cells were used as APCs, and the optimum concentations of aGalCer and the sulfatides used were determined in preliminary experiments. Similar responses were obtained for a 100 ng/ml dose of aGalCer and a 50 mg/ml dose of C16:0 (Fig. 3A), indicating that aGalCer stimulates the proliferation of CD4^+^ T cells much more efficiently than C16:0. Moreover, at each dose of sulfatide tested, C16:0 was more stimulatory than C24:0, particularly at a 100∶1 ratio of CD4^+^:CD4^–^ T cells. Thus, the rank order of stimulation of CD4^+^ T cell proliferation *in vitro* is aGalCer > C16:0>C24:0. It follows that the greater ability of C24:0 sulfatide to protect from T1D does not correlate directly with its relative capacity to stimulate a CD4^+^ T cell proliferative response.

Since the presence of C24:1 sulfatide is enriched in islet beta cells, we examined whether activation of the T cell population reactive to this self-glycolipid can influence the course of spontaneous T1D in NOD mice. Interstingly, a significant spleen T cell proliferative response to sulfatide was obtained in female diabetic or non-diabetic mice following an *in vitro* stimulation. Notably, the responses to sulfatide in male diabetic NOD mice, which exhibit a lower disease frequency than female NOD mice, are about 10-fold lower than in females (Fig. 3B, right panels). On the other hand, the responses to aGalCer are essentially identical in both male and female mice at the concentrations examined (Fig. 3B, left panels). Spleen T cell proliferative responses to another myelin-derived lipid, GM1, were not detected (our unpublished data). Interestingly, the proliferative responses of spleen T cells to sulfatide in NOD mice are more pronounced than those previously determined in non-T1D susceptible C57BL/6, SJL/J or B10.PL mice [2], [9]. Similar to T cell responses in other mouse haplotypes, the T cell response to sulfatide is blocked in the presence of an anti-CD1d mAb and is absent in NOD.CD1d^−/−^ mice.

### Different CD4^+^ T Cell Cytokine Secretion Profiles Induced by C16:0 and C24:0 may Partially Account for Why C24:0 Yields More Protection from T1D than C16:0

**Figure 4:**
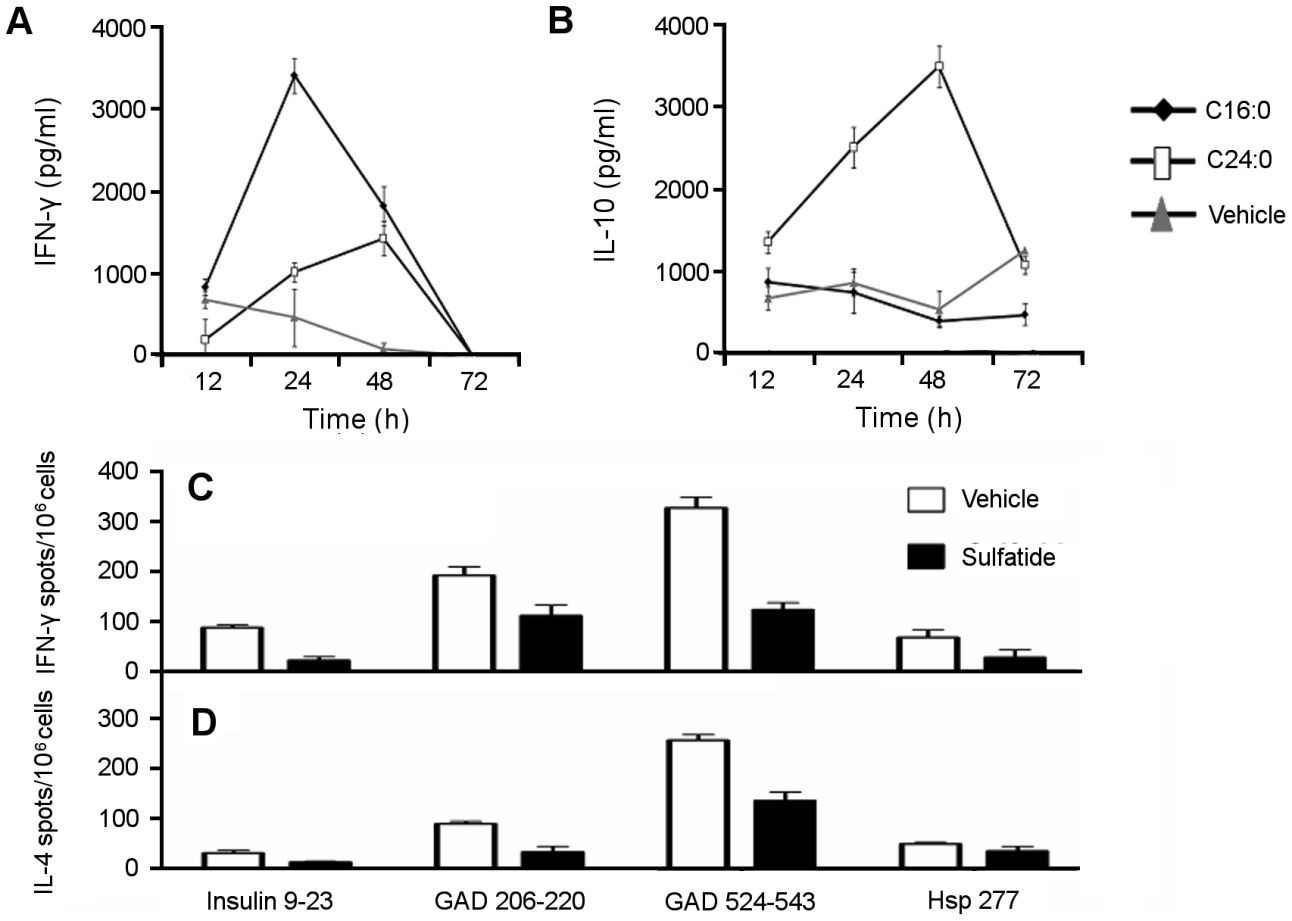
Analyses of NOD T cell cytokine secretion responses induced by sulfatides. (**A**, **B**) C16:0 and C24:0 sulfatide stimulate different CD4^+^ T cell cytokine secretion profiles. NOD spleen CD4^+^ T cells were co-cultured for 72 h with mitomycin treated CD4^–^ cells at a CD4^+^:CD4^–^ ratio of 100∶1 in the presence of control vehicle or sulfatide (C16:0 or C24:0, 50 mg/ml), as described in Fig. 3A. The concentration of IFN-g (A) and IL-10 (B) secreted into cell supernatants were analyzed by ELISA. IL-2 and IL-4 were not detected in these supernatants. Data shown were obtained from one of three representative and reproducible experiments. (**C**, **D**) Treatment with sulfatide inhibits the induced anti-islet diabetogenic T cell cytokine responses. Female NOD mice (4 week-old, 4 mice/group) were injected i.p. with sulfatide (20 mg/mouse) or vehicle/PBS. One week later, the treated mice were immunized with 100 mg of either the insulin p9–23, GAD206-220, GAD524-543 or hsp277 peptide. Ten days following antigenic challenge, PLN lymphocytes were assayed for their proliferative ([^3^H]-thymidine incorporation) and cytokine (IFN-g, IL-4) secretion responses (Elispot assay). The average frequencies of IFN-γ (**C**) or IL-4 (**D**) secreting cells reactive to different islet antigens are shown. In comparison to control vehicle values, statistically significant reductions in the frequencies of cytokine-secreting islet antigen-reactive T cells were found for the insulin and GAD peptides but not Hsp peptide, as follows. Insulin 9–23 (P = 0.0001 for IFN-g, P = 0.0078 for IL-4); GAD 206-220 (P = 0.012 for IFN-g, P = 0.0018 for IL-4); GAD 524-543 (P<0.0001 for IFN-g, P = 0.0328 for IL-4); and Hsp 277 (Not significant, P = 0.0542 for IFN-g; Not significant, P = 0.0952 for IL-4).

Our finding that protection from T1D by C24:0 does not correlate with its capacity to stimulate a more vigorous CD4^+^ T cell proliferative response than C16:0 raised the possibility that the differential responsiveness of CD4^+^ T cells to these two sulfatide isoforms depends more on their cytokine secretion profile. To test this possibility, we performed a comparative kinetic analysis of cytokine secretion by NOD splenic CD4^+^ T cells stimulated at a CD4^+^ T cell:APC (CD4^–^ T cell) ratio of 100∶1 *in vitro* for varying times (12–72 h) with either control vehicle or an optimum dose (50 mg/ml) of C16:0 or C24:0. Interestingly, C16:0 stimulated significantly higher secretion of IFN-g than C24:0, particularly at the peak of the C16:0 response at 24 h (Fig. 4A). Both the C16:0 and C24:0 induced responses returned rapidly to base line levels by 72 h. In contrast, C24:0 elicited maximal secretion of IL-10 at 48 h, whereas C16:0 did not stimulate any significant IL-10 secretion above that observed for the control vehicle at any time point (Fig. 4B). The levels of IL-2 and IL-4 secretion detected in the C16:0 and C24:0 stimulated responses were also not significant above those observed for the control vehicle (our unpublished data). Thus, since IFN-g and IL-10 are pro-inflammatory and anti-inflammatory cytokines, respectively, these cytokine secretion responses may explain in part why C24:0 is more protective from T1D than C16:0.

### Sulfatide Treatment Inhibits Islet Antigen Reactive Diabetogenic T Cell Proliferative and Cytokine Responses in NOD Mice

Previously, we found that in different mouse models of EAE, the proliferation and cytokine secretion of myelin basic protein or proteolipid-reactive encephalitogenic CD4^+^ T cells is significantly diminished following immune regulation mediated by sulfatide-reactive T cells [2, Maricic et al, in preparation]. Thus, in this study, we investigated whether the cytokine secretion profiles of T cells reactive to any of the dominant determinants from candidate islet antigens shown to be involved in the diabetogenic process are also influenced after sulfatide administration in NOD mice.

After i.p. injection of sulfatide (20 mg/mouse), NOD mice were challenged with different diabetogenic peptides derived from the islet antigens glutamic acid dehydrogenase (GAD), heat shock protein 60 (hsp60), or insulin. Ten days after antigenic challenge, PLN T cell proliferative and cytokine responses to the respective antigen were analyzed. The frequency of both IFN-g and IL-4-secreting T cells in Elispot assays of responses to the insulin 9–23, GAD206-220, GAD524-543 and hsp277 peptides were significantly decreased in sulfatide-treated mice (Fig. 4, C and D). These data support the notion that activation of sulfatide-reactive type II NKT cells results in the inhibition of effector T cells that mediate the onset of T1D in NOD mice.

**C16:0 and C24:0 Sulfatide do not Stimulate iNKT Cell Early Activation and Expansion**

**Figure 5:**
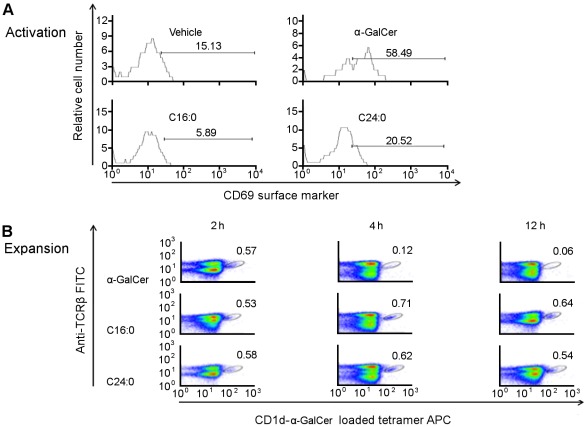
Analyses of sulfatide-induced stimulation of the activation and expansion of type I iNKT cells. (**A**) C16:0 and C24:0 do not stimulate the activation of type I iNKT cells. At 2 h after female NOD mice (3–5 week-old, n = 5/group) were injected (i.p.) with vehicle (PBS), aGalCer (5 mg) or sulfatide (C16:0 or C24:0, 100 mg), their splenocytes were analyzed by FACS for the surface expression of the early activation surface marker CD69 on TCRβ^+^a-GalCer/CD1dTet^+^ iNKT cells. (**B**) C16:0 and C24:0 do not stimulate the expansion of type I iNKT cells. At 2 h, 4 h and 12 h after female NOD mice (3–5 week-old, n = 5/group) were injected (i.p.) with either control vehicle (PBS), aGalCer (4 mg) or sulfatide (C16:0 or C24:0, 100 mg), splenocytes were analyzed by FACS for the expansion of TCRb^+^a-GalCer/CD1dTet^+^ iNKT cells. Data shown in (**A**) and (**B**) were obtained from one of two representative and reproducible experiments.

Our observation that C16:0 and C24:0 activated donor NOD CD4^+^ T cells differ in their level of protection from T1D conferred upon transfer to NOD.*Scid* recipients (Fig. 1C) raised the possibility that these two sulfatide isoforms may vary in their capacity to activate and expand CD4^+^ T cells, and type II NKT cells in particular, as sulfatide is a ligand for type II NKT cells but not type I iNKT cells [2], [5], [9], [32]. Initially, we obtained αGalCer/CD1d tetramers to monitor the activation and expansion of type I iNKT cells. In the following studies, NOD mice were treated i.p. *in vivo* with a dose of sulfatide (100 mg) or aGalCer (4 mg) that yielded optimum protection from the transfer of T1D. At 2 h post-injection of NOD mice (n = 5/group, 3–5 week-old) with vehicle, sulfatide (C16:0 or C24:0) or aGalCer, splenocytes were analyzed by FACS for the frequency of iNKT cells (gated TCRβ^+^aGalCer/CD1d Tet^+^ cells) that express the CD69 early activation surface marker. Compared to vehicle-stimulated iNKT cells, CD69 surface expression was increased about 4-fold on aGalCer stimulated iNKT cells while no significant increase in CD69 expression was detected on iNKT cells exposed to C16:0 or C24:0 (Fig. 5A). Similar results were obtained when a higher dose (200 mg) of C16:0 or C24:0 was used.

Next, we monitored the kinetics of iNKT cell expansion in the spleen and PLN, two sites of activation of iNKT cells that mediate protection from T1D, using the same protocol as described for the iNKT cell expansion studies discussed above. While the frequency of aGalCer stimulated iNKT cells in the spleen was about 0.6% at 2 h post-injection (control vehicle induced a frequency of 0.2%), this frequency decreased significantly by 4 h and 12 h after injection (Fig. 5B), consistent with the kinetics of down-regulation of iNKT cell TCR surface expression previously reported [33]. Interestingly, although a similar low percentage (0.5%–0.6%) of iNKT cells was detected at 2 h post-injection of C16:0 or C24:0 sulfatide, the decrease in frequency and down-regulation of TCR surface expression on iNKT cells was not seen at 4 h and 12 h post-injection. Similar results were obtained for CD4^+^ T cells and iNKT cells in the PLN of the sulfatide treated mice (data not shown). Thus, unlike aGalCer, C16:0 and C24:0 sulfatide do not stimulate the expansion or early activation of iNKT cells, consistent with the ability of sulfatide to function as a ligand for type II NKT but not type I iNKT cells.

### Enrichment of Sulfatide-reactive Type II NKT Cells in Draining Pancreatic Lymph Nodes

**Figure 6:**
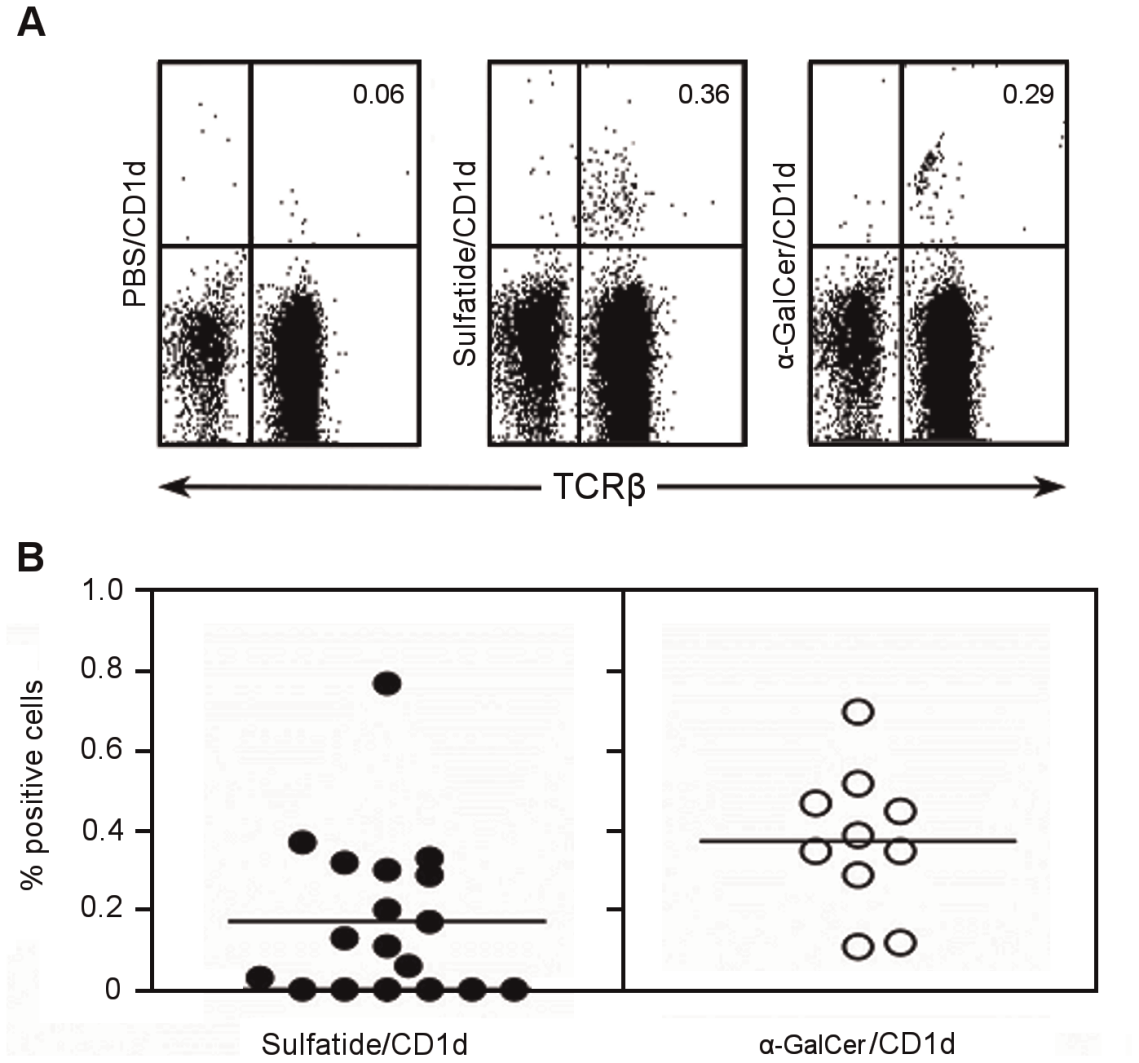
Sulfatide-reactive Type II NKT cells are enriched in the PLN during the development of T1D. PLN lymphocytes isolated from the pancreas of female NOD mice (6–20 week-old) were stained with sulfatide/CD1d-, aGalCer/CD1d- or unfilled PBS/CD1d-tetramers and anti-TCRb and then analyzed by flow cytometry. (**A**) The numbers shown in each panel indicate the % tetramer^+^ cells. (**B**) The scatter plots show the mean values of sulfatide/CD1d-tetramer^+^ (0.17%) and αGalCer/CD1d-tetramer^+^ (0.37%) cells in diabetic NOD mice (6–20 week-old). Note that we were not able to detect any correlation between NKT cell numbers and the severity of T1D, as we did not find any significant correlation between the frequency of sulfatide-CD1d-tetramer^+^ cells in diabetic vs. non-diabetic NOD mice.

In wild-type mice, sulfatide-reactive type II NKT cells have a similar tissue distribution to that of type I iNKT cells [2], [9], [19]. Thus, sulfatide/CD1d-tetramer^+^ T cells are localized predominantly in the liver, and only a minor subpopulation (∼0.1%) is present in the spleen. In NOD mice, sulfatide/CD1d-tetramer^+^ cells are barely detectable in the spleen at any age (6–20 weeks) examined and irrespective of the onset of T1D. Since sulfatide is highly enriched in the membranes of islet beta cells, we reasoned that it may be presented as an antigen to type II NKT cells localized in the PLN during the development of destructive insulitis in diabetic NOD mice. In this scenario, the frequency of sulfatide/CD1d-tetramer^+^ cells should be enriched in the draining PLN. To determine whether sulfatide-reactive type II NKT cells accumulate in the draining PLN during the onset of T1D, PLN-derived mononuclear cells isolated from NOD mice (aged 6–20 weeks) were stained with either sulfatide/CD1d- or αGalCer/CD1d-tetramers for the identification of type II and type I NKT cells, respectively. We found that sulfatide-reactive T cells are present at a significantly higher frequency (0.36%) of infiltrating T cells in the PLN than in the liver or spleen (<0.1%) (Fig. 6). Interestingly, aGalCer-reactive type I NKT cells are also present in the PLN (0.29%) but are not enriched as much as in the liver where they constitute a major subpopulation (>10%). We detected a similar enrichment of sulfatide-CD1d-tetramer^+^ T cells in the CNS tissue of B10.PL mice during MBP-induced EAE [2]. Enrichment of sulfatide/CD1d-tetramer^+^ cells was not found in other organs, including the liver and spleen and their frequency remained <0.1%. Collectively, these data suggest that sulfatide-reactive type II NKT cells may accumulate in those organs where a destructive immune response of sulfatide-enriched membranes occurs during an inflammatory response.

## Discussion

Recent studies have shown that the restoration of an iNKT cell deficiency in NOD mice following their activation with aGalCer protects against T1D [3], [4]. Protection against T1D by CD1d-restricted type II NKT cells in transgenic mice has also been demonstrated [18], [32]. While aGalCer is not recognized by type II NKT cells, sulfatide, a b-linked self-glycolipid, can stimulate the expansion of type II NKT cells [2], [9]. Here, we show that the longer C24:0 but not the shorter C16:0 sulfatide isoform can induce NKT cells to transfer the delay of onset of T1D (Fig. 1C), and that this delay is CD1d-restricted as T1D was not prevented in sulfatide treated CD1d-deficient NOD mice (Fig. 1B). The latter result and the inability of both sulfatide isoforms to activate and expand iNKT cells (Fig. 5) suggest that type II NKT cells may play a regulatory role in delay/protection from T1D. This idea is consistent with the known anti-inflammatory properties of sulfatide such as the reduced secretion of pro-inflammatory cytokines and chemokines [28], [34] and inhibition of proliferation of autoreactive T cells from patients with T1D [35]. Furthermore, the accumulation of sulfatide-reactive type II NKT cells but not type I NKT cells in the PLN of sulfatide-treated NOD mice suggest that these sulfatide stimulated type II NKT cells may regulate susceptibility to T1D (Fig. 6).

Previously, we reported that brain-derived sulfatide can mediates protection against T1D [29], but the mechanism of protection and CD1d-dependence was not investigated. Moreover, as sulfatide is comprised of both the C16:0 and C24:0 isoforms, it remained to determine whether one or both isoforms protects from T1D. In this study, we analyzed the ability of sulfatide C16:0 and C24:0 to stimulate CD4^+^ T cells from NOD mice to transfer protection from T1D. Prior analyses of stimulation of CD4^+^ T cell *in vitro* proliferative responses showed that a robust response to sulfatide C16:0 and C24:0 required about a 500-fold higher concentration (50 mg/ml) of C16:0 or C24:0 than that of aGalCer (100 ng/ml) (Fig. 3A). Therefore, in our adoptive transfer of T1D studies, we treated NOD mice with a high dose (100 mg) of C16:0 or C24:0 sulfatide equivalent to that reported previously for brain sulfatide induced protection from T1D [29]. Efficient protection from T1D was provided by administration of high dose (100 mg/dose) C24:0 sulfatide and low dose (4 mg/dose) aGalCer, but not by high dose (100 mg/dose) C16:0 sulfatide. Thus, this result identifies C24:0 as the sulfatide isoform that can stimulate NKT cells to delay the transfer of T1D. Importantly, neither brain sulfatide [35] nor its C24:0 sulfatide isoform was found to activate or expand iNKT cells (Fig. 5), further supporting the role of type II NKT cells in C24:0 induced protection from T1D.

Cytokines produced by glycolipid stimulated iNKT cells can regulate the phenotype and function of interacting APCs [3], [19], [36]. Our analyses of the cytokine secretion profiles of CD4^+^ T cells stimulated with a high dose of C16:0 or CD24:0 sulfatide demonstrated a more rapid and preferential secretion of IFN-γ secretion by C16:0 stimulated CD4^+^ T cells. The latter C16:0 induced response peaked at 24 h post stimulation. In contrast, the IFN-g response of C24:0 stimulated cells did not peak until 48 h post-stimulation, and this level of secretion was about 3-fold lower than that obtained for the C16:0 induced response at 24 h. Importantly, C24:0 sulfatide stimulated cells secreted large amounts of IL-10 and reached a maximal response at 48 h post-stimulation at which time the level of IL-10 secretion by C16:0 stimulated T cells was not significantly different from the control vehicle response. These results implicate a role for IL-10 in C24:0 sulfatide-mediated protection of NOD mice against T1D. IL-10 is an anti-inflammatory cytokine that elicits immunosuppression, and when expressed in the pancreas, IL-10 mediates elevated regulatory T (Treg) cell activity and protection from T1D [37]. Repeated treatment of mice with aGalCer reduces the ability of iNKT cells to secrete IFN-g while retaining the same level of IL-10 production [31], and IL-10 treated DCs become tolerogenic and induce anergy in iNKT cells [37]. Tolerogenic DCs induced by anergic iNKT cells in turn are characterized phenotypically by their decreased IL-12 and increased IL-10 secretion responses [38]. Our recent results obtained with a C16:0 fatty acyl chain structural derivative of aGalCer suggest that tolerogenic DCs in the PLN may suppress autoreactive T cells responsible for islet beta cell death and also generate IL-10 dependent Th2 and Treg cell responses that aid in protection against T1D [39]. This result further emphasizes the important role that IL-10 may play in protection from T1D. Accordingly, we find that the frequency of diabetogenic Th1-like effector T cells reactive to islet antigens is also significantly reduced in mice treated with sulfatide (Fig 4). These data suggest that sulfatide treatment results in not only the induction of anergy in type I NKT cells but also the inhibition of islet protein antigen-reactive MHC class II-restricted pro-inflammatory T cells.

The capacity of sulfatide reactive type II NKT cells to anergize type I iNKT cells prevents inflammatory liver disease in mice by modulating the function of DCs [19]. In this study, we found that C24:0 sulfatide stimulated CD4^+^ T cells undergo reduced proliferation (Fig. 3A) and increased secretion of IL-10 (Fig. 4B). These results suggest that C24:0 stimulated type II NKT cells may modulate the ability of DCs to secrete IL-10, and thereby suppress the activation and expansion of type I iNKT cells. Thus, interaction between type II NKT cells, DCs and type I iNKT cells may be one of several pathways of immune cell crosstalk that can be regulated by C24:0 sulfatide and lead to protection from T1D [15]. This proposed pathway of immune cell crosstalk is also consistent with a very recent report that CD4^+^ type II NKT cells mediate ICOS and programmed death-1–dependent regulation of diabetogenic CD4^+^ T cells and protection from T1D [40].

The fatty acyl chain length of a lipid can determine its ability to stimulate the proliferation and function of both CD1d-restricted type I NKT cells [30], [31], [39], [41]–[47] and CD1d-restricted type II NKT cells [32]. Interestingly, sulfatide C24:1, a major component of the native sulfatide mixture in brain myelin, may be an endogenous ligand for type II NKT cells activated during demyelinating inflammatory diseases of the central nervous system [32].

C24:1 and particularly its lyso C18:1 form (lacks a fatty acyl side chain) potently stimulate type II NKT cells. In contrast, sulfatide isoforms with either a shorter fatty acyl chain length (≤ C18:0) or saturation of the long fatty acyl chain (C24:0) are weak stimulators of type II NKT cells, and fatty acid hydroxylation abolishes the response [32], [41], [42]. Compared to sulfatide isoforms in brain, sulfatide isoforms in pancreatic beta cells contain more short fatty acyl chains, e.g. C16:0, and lack hydroxylation [1], [39]. Based on data obtained with type II NKT hybridoma cells *in vitro*, one would expect C24:0 and C16:0 to be weak stimulators of type II NKT cells *in vivo*, which is precisely what we observed with lymphocytes from NOD mice. However, despite the equal amount of C16:0 and C24:0 in islet beta cells, we unexpectedly found that C24:0 induced more efficient delay of transfer of T1D than C16:0. This might be attributed to the ability of C24:0 to elicit the reduced proliferation and elevated IL-10 secretion of CD4^+^ T cells, in accordance with our recent studies of the C20:2 N-acyl variant of aGalCer. C20:2 activates NOD type I iNKT cells more weakly than αGalCer, and iNKT cells activated *in vivo* with C20:2 enter into and exit from anergy more rapidly than after activation by aGalCer [31], [41]. Importantly, this shorter duration of iNKT cells in the anergic state promotes the more rapid induction of tolerogenic DCs in an IL-10 dependent manner, gives rise to reduced iNKT cell death, and enables C20:2 stimulated iNKT cells to elicit enhanced protection from T1D. It remains to determine whether C24:0 sulfatide activates type II NKT cells to enter into and exit from anergy more rapidly than C16:0 activation and thereby yield less type II NKT cell death and increased protection from T1D.

In conclusion, our findings demonstrate that while bovine brain-derived sulfatide consists of several molecular species that vary in fatty acid chain length, unsaturation and hydroxylation, treatment of NOD mice with the long fatty acid chain sulfatide isoform results in the activation of type II NKT cells that may mediate significant protection from T1D. Consistently, long fatty acid sulfatide isoforms, including C24:1 and C24:0, are immunodominant in mice and can bind with high avidity to murine CD1d molecules [2], [9]. Furthermore, treatment with these isoforms can also control other autoimmune diseases, including antigen-induced chronic and relapsing EAE (2, and *Maricic et al., in preparation*), concanavalin A-induced hepatitis [19] and hepatic ischemic reperfusion injury [21]. Collectively, these studies may lead to novel strategies for the use of sulfatide isoforms to therapeutically intervene in clinical trials of T1D.

## Materials and Methods

### Mice

NOD/Del, NOD.Scid and NOD.CD1d−/− mice were bred and maintained in a specific pathogen-free barrier facility under the direction of the Animal Care and Veterinary Services at the Robarts Research Institute, Western University (London, ON, Canada) and according to the Canadian Council for Animal Care guidelines. NOD mice were also purchased from The Jackson Laboratory/Taconic Farms and kept under specific pathogen-free conditions in our colony at the Torrey Pines Institute for Molecular Studies (San Diego, CA). These experiments were performed in compliance with federal and institutional guidelines and have been approved by the Institutional Animal Care and Use Committee of the Torrey Pines Institute for Molecular Studies.

The Animal Care and Veterinary Services Committee at Western University specifically approved this study (protocol number 2008-025). The incidence of T1D in female NOD mice in our colony is ≥80% by 30 weeks.

### Monitoring of T1D

Mice were monitored for hyperglycemia beginning at 15 weeks of age in the spontaneous model and at 8 weeks of age in the adoptive transfer model by measurement of BGL twice weekly, as described. Mice were considered diabetic when two consecutive BGL readings of >11.3 mmol/l were obtained.

### Glycolipids, Sulfatide and Antibodies

Synthetic KRN7000 (aGalCer, C26:0/C18:0) was kindly provided by Kirin Pharmaceutical Research Laboratories (Gunma, Japan), solubilized in water and injected i.p. into mice (4 mg/dose). Native sulfatide was isolated from pig brain as described [48] or purchased from Matreya Inc. (2). Sulfatide C24:0 and C16:0 isoforms were prepared by semi-synthesis [48], [49] or acquired as before (2). Allophycocyanin (APC)-conjugated PBS-57-loaded and -unloaded CD1d tetramers for staining mouse iNKT cells were provided by the NIH Tetramer Core Facility (Emory University. Fluorescein isothiocyanate (FITC)-conjugated anti-TCRb (H57–597), anti-CD4 (GK1.5), anti-CD8 (53–6.7), and anti-CD3e (eBio500A2) mAbs as well as and phycoerythrin (PE)-conjugated anti-CD69 (H1.2F3), anti-IL-4 (11B11), anti-IFN-γ (XMG1.2), anti-IL-12 (C17.8), anti-IL-10 (JES5-16E3) and anti-IgG2b (eB149/1OH5) mAbs were purchased from eBiosciences or BD Biosciences.

### Cell Suspensions and Adoptive Transfer Model of T1D

The aGalCer and sulfatide (C24:0 or C16:0 isoform) glycolipids (100 mg) were suspended in PBS [49] and injected i.p. into female NOD (6–8 week-old) mice on day 0 and day 4. Spleens and pancreatic lymph nodes (PLN) were harvested after 1 week of rest. Single-cell suspensions of splenocytes (10×10^6^) and PLN lymphocytes (0.5×10^6^) were prepared in PBS containing 2% FBS (PBS/FBS) [50], and were co-injected into female (3 week-old) NOD.*Scid* mice (n = 10/treatment group).

### Analyses of Cell Proliferation and Cytokine Secretion

Spleen- and PLN-derived lymphocytes (5×10^5^ cells for proliferation analysis, 5×10^6^ cells for cytokine analysis) were cultured (72 h, 37°C) in triplicate with glycolipid (100 ng/ml) or vehicle, as described [50]. To assay proliferation, cells were pulsed with [^3^H]-thymidine (1 mCi/well, Perkin Elmer) for the last 18 h of culture, harvested and incorporated radioactivity was quantified using a 1450 Microbeta scintillation counter (Perkin Elmer). All cultures were maintained in complete RPMI (RPMI 1640 medium supplemented with 10% heat-inactivated FBS (Hyclone), 1 mM sodium pyruvate, 10 mM HEPES, 100 U/ml penicillin, 100 mg/ml streptomycin, 2 mM L-glutamine, and 0.05 mM 2-ME (Invitrogen Life Technologies). A standard sandwich ELISA was performed to analyze mouse cytokines in culture supernatants using paired antibody kits for IL-4, IL-10, IL-2, IFN-g (BD Biosciences). Streptavidin-horse radish peroxidase (HRP) conjugate and development solution for BD OptiEIA Reagent Set A (BD Bioscience) were used for signal detection at a dual wavelength of 450/570 nm using a Benchmark Microplate Reader (Bio-Rad).

The frequency of IFN-g and IL-4-producing cells were enumerated by cellular ELISPOT essentially as described earlier [19]. In brief, splenocytes (5×10^6^ cells/ml) were cultured for 48 h in 24-well plates either with medium alone or with different islet antigen-derived peptides (20 mg/ml). Nitrocellulose plates (Millipore) were coated overnight at 4°C with anti-IFN-g or anti-IL-4 Abs. After blocking the coated plates, Ag-stimulated cells were added at graded concentrations for 24 h at 37°C. The wells were then incubated with biotin-conjugated anti-IFN-g or anti-IL-4 mAbs followed by incubation with avidin peroxidase (Vector Laboratories). Spots were developed by the addition of 3-amino-9-ethylcarbazole substrate (Sigma-Aldrich), and were counted using a computerized image analysis system and the image analyzer program, NIH Image 1.61.

### Isolation of CD4^+^ T Cells

A mouse CD4^+^ T cell isolation kit (Miltenyi Biotec) was used to isolate CD4^+^ splenocytes by negative selection with a purity of 93%, as assayed by flow cytometry. The CD4^–^ T cells (15×10^6^/ml in PBS/FBS) obtained were treated (37°C, 20 min) with mitomycin C (100 ml/ml) and used as a source of APCs in proliferative responses.

### Flow Cytometry

Single-cell suspensions of splenocytes and PLN lymhocytes were treated (15 min, 4°C) with an anti-FcgR mAb and then stained with an FITC-anti-TCR mAb (H57–597) and APC-labeled empty or PBS-57-loaded CD1 tetramers (provided by NIH Tetramer Core Facility), PE CD69 (H1.2F3), and PE-rat IgG2b isotype control (eB149/10H5). Sulfatide/CD1d-tetramers were generated as described earlier (2). Flow cytometry was performed using FACSCalibur and CellQuest software (BD Biosciences) or FACSCanto II and FACSDiva software. Analyses were conducted using FlowJo software (Treestar, Ashland, OR). Intracellular cytokine (IL-4, IFN-g, IL-12) staining was performed using a BD Cytofix/Cytoperm buffer set (BD Biosciences) [50].

### Statistical Analyses

Results are expressed as mean ± standard error of the mean (SE). Statistical analyses were performed using the Student’s t test or log rank test (for T1D incidence) (Prism, version 4.0, GraphPad software). Differences were considered statistically significant at P values ≤0.05.
