# Correction: NKT Cells Stimulated by Long Fatty Acyl Chain Sulfatides Significantly Reduces the Incidence of Type 1 Diabetes in Nonobese Diabetic Mice

**DOI:** 10.1371/annotation/b5c4c567-f17b-4ebb-a6ea-d3cdff36d5ce

**Published:** 2012-06-15

**Authors:** Lakshmimathy Subramanian, Hartley Blumenfeld, Robert Tohn, Dalam Ly, Carlos Aguilera, Igor Maricic, Jan-Eric Mansson, Karsten Buschard, Vipin Kumar, Terry L. Delovitch

There is information missing from the Funding section. The complete funding information is:

This work was supported by the Juvenile Diabetes Research Foundation (JDRF) grants 24-2007-388 (TLD) and 24-2007-362 (VK). Additional support was provided by the Canadian Institutes of Health Research grant MOP 64386 (TLD) and the National Institutes of Health grant R01 CA100660 (VK). L. Subramanian was the recipient of Summer Research Training Program Studentship from the University of Western Ontario, H. Blumenfeld was the recipient of a Schulich Graduate Enhancement Scholarship and Department of Microbiology and Immunology Graduate Entrance Fellowship, and R. Tohn was the recipient of a Schulich Graduate Enhancement Scholarship, Department of Microbiology and Immunology Graduate Entrance Fellowship, and Ontario Graduate Scholarship. The funders had no role in study design, data collection and analysis, decision to publish, or preparation of the manuscript. 

