# Correction: NKT Cells Stimulated by Long Fatty Acyl Chain Sulfatides Significantly Reduces the Incidence of Type 1 Diabetes in Nonobese Diabetic Mice

**DOI:** 10.1371/annotation/b737472b-f38a-4ef6-b10c-e052c3b66148

**Published:** 2012-07-10

**Authors:** Lakshmimathy Subramanian, Hartley Blumenfeld, Robert Tohn, Dalam Ly, Carlos Aguilera, Igor Maricic, Jan-Eric Mansson, Karsten Buschard, Vipin Kumar, Terry L. Delovitch

There is a typographical error in the title. The correct title is: NKT Cells Stimulated by Long Fatty Acyl Chain Sulfatides Significantly Reduce the Incidence of Type 1 Diabetes in Nonobese Diabetic Mice. The correction citation is: Subramanian L, Blumenfeld H, Tohn R, Ly D, Aguilera C, et al. (2012) NKT Cells Stimulated by Long Fatty Acyl Chain Sulfatides Significantly Reduce the Incidence of Type 1 Diabetes in Nonobese Diabetic Mice. PLoS ONE 7(5): e37771. doi:10.1371/journal.pone.0037771 

